# Chemical composition and antibacterial activity of the essential oils from *Launaea resedifolia *L

**DOI:** 10.1186/2191-2858-2-2

**Published:** 2012-01-20

**Authors:** Amar Zellagui, Noureddine Gherraf, Segni Ladjel, Samir Hameurlaine

**Affiliations:** 1 Laboratory of Biomolecules and Plant Breeding, Life Science and Nature Department, University of Larbi Ben Mhidi Oum El Bouaghi, Algeria; 2 Kasdi Merbah University, Ouargla, Algeria

**Keywords:** chemical composition, antibacterial activity, essential oils, *Launaea resedifolia*

## Abstract

**Background:**

Several species of the genus *Launaea *are used in folk medicine such as in bitter stomachic, skin diseases, and reported to have antitumor, insecticide, and cytotoxic activities. The antimicrobial activities of coumarin constituents and the neuropharmacological properties have been investigated as well. In this study, the chemical composition of essential oils from *Launaea resedifolia *L. has been identified using the ordinary GC-MS technique to reveal the presence of 19 compounds dominated by dioctyl phthalate. Moreover, the antibacterial activity of the crude oil has been carried out using disk diffusion method against seven bacteria strains.

**Results:**

Nineteen compounds of essential oil of *L. resedifolia *L. were identified, representing 86.68% of the total oil. The compounds were identified by spectral comparison to be mainly esters, alcohols, ketones, and terpenes. The principal constituents are dioctyl phthalate (39.84%), Decanoic acid, decyl ester (12.09%), 11-Octadecenal (11.24%), and Eucalyptol (07.31%), while others were present in relatively small amounts. As far as antibacterial essays are concerned, it was found that the oils are active against most of the tested bacterial strains.

**Conclusion:**

A major constituent in visible parts was Dioctyl phthalate (39.84%) and the yield of essential oils was 0.9%. These extracts reveal *in vitro *antibacterial activity on the studied bacterial, confirmed by the inhibition zone diameter ranging from 11 to 37 mm and a MIC value between 0.09 and 0.69 depending on the microorganism being tested.

## 1. Background

The genus *Launaea *(Asteraceae) is represented in the flora of Algeria by nine species, namely, *Launaea acanthoclada, Launaea angustifolia, Launaea anomala, Launaea arborescens, Launaea cassiniana, Launaea glomerata, Launaea nudicaulis, Launaea quercifolia*, and *Launaea resedifolia *[1,2]. *L. resedifolia *(local name "laadid, Azim") is a perennial herb widely distributed in the arid regions of Mediterranean area, where it is abundant in south east of Algeria.

Several species of this genus are used in folk medicine in bitter stomachic, skin diseases, and reported to have antitumor, insecticide and cytotoxic activities [3]. The antimicrobial activities of coumarin constituents [4] and the neuropharmacological properties [5] have been investigated as well.

To the best of the authors' knowledge, there are no reports about the chemical content and biological effect of the essential oils of *L. resedifolia*. There no reports on the essential oils of the species of the genus launaea except that reported by Cheriti et al. [6]. In continuation of our phytochemical and antibacterial studies of the Algerian Sahara medicinal plants [7-11], we report here the findings of our studies on the composition and antimicrobial activity of *L. resedifolia *essential oils. The species was collected during the flowering stage in south-eastern Algeria (Ouargla) and identified by Dr. Abdelmadjid Chahma, Biology Department, Ouargla University, Algeria. A voucher specimen was deposited at the herbarium under the code NG 27.

## 2. Results and discussions

The aerial parts of *L. resedifolia *were collected in March 2010 in the outskirts of Ouargla (600 km south of Algiers). The plant was identified by Dr. Abdelmadjid Chahma. A voucher specimen was deposited at the herbarium under the number NG 27.

### 2.1 Isolation of essential oils

An aliquot of 200 g of the visible parts of *L. resedifolia *was cut into pieces, air-dried under shade, and subjected to hydrodistillation on a Clavenger-type apparatus for 4 h. The distillate was then extracted using diethyl ether. The resulting extract was dried on anhydrous sodium sulphate. Diethyl ether was removed carefully and the essential oil was collected and stored at 4°C until analysis. The oil yield was calculated relative to the dry matter.

### 2.2 GC-MS analysis

The oil was analyzed by GC/MS using a Agilent 5973EI mass selective detector coupled with an Agilent GC6890A gas chromatograph, equipped with a cross linked 5% PH ME siloxane HP-5MS capillary column (30 m × 0.25 mm, film thickness 0.25 μm). Operating conditions were as follows: carrier gas, helium with a flow rate of 1 mL/min; column temperature 50°C for 1 min, 50-150°C (3°C/mn), 150-250°C (5°C/mn) then isothermal for 5 min.

Injector and detector temperatures: 280°C; split ratio, 1:50.

The MS operating parameters were as follows: ionization potential, 70 eV; ionization current, 2 A; ion source temperature, 200°C; resolution, 1000.

### 2.3 Identification of components

Identification of oil components was achieved on the basis of their retention indices (RI) (determined with reference to a homologous series of normal alkanes), and by comparison of their mass spectral fragmentation patterns with those reported in the literature [12] and stored on the MS library (NIST database). The concentration of the identified compounds was computed from the GC peak total area without any correction factor.

### 2.4 Antibacterial activity

In recent years due to an upsurge in antibiotic-resistant infections, the search for novel archetype prescriptions to fight infections is an absolute necessity and in this regard, plant essential oils may offer a great potential and hope. Several studies have reported the efficacy of antibacterials obtained from the essential oils of various plant species [13-15]. In this study, antibacterial activity of essential oil extracted from aerial parts of *L. resedifolia *was tested using different bacterial strains: *Escherichia coli, Staphylococcus aureus, Staphylococcus intermedius, Proteus mirabilis, Streptococcus pyogenes, Pseudomonas aeruginosa*, and *Klebsielle pneumoniae*. In addition, the composition of volatile compounds was also determined.

All bacterial samples were obtained from the bacteriology laboratory SAIDAL, Algeria. The antimicrobial activity tests were carried out using disk diffusion method [15] against seven human pathogenic bacteria, including Gram positive and Gram-negative bacteria. The bacteria strains were first grown on Muller Hinton medium at 37°C for 24 h prior to seeding on to the nutrient agar.

A sterile 6-mm diameter filter disk (Whatman paper n^o ^3) was placed on the infusion agar seeded with bacteria, and each extract suspended in water was dropped on to each paper disk (40 μL per disk). The treated Petri disks were kept at 4°C for 1 h, and incubated at 37°C for 24 h. The antibacterial activity was assessed by measuring the zone of growth inhibition surrounding the disks. Each experiment was carried out in triplicate [16].

The minimal inhibitory concentration (MIC) was determined by dilution of the essential oil in dimethyl sulphoxide (DMSO) pipetting 0.01 mL of each dilution onto a filter paper disc [17,18]. Dilutions of the oil within a concentration range of 10-420 g/mL were also carried out. MIC was defined as the lowest concentration that inhibited the visible bacterial growth.

A negative control was also included in the test using a filter paper disk saturated with DMSO to check possible activity of this solvent against the bacteria assayed. The experiments were repeated three times.

### 2.5 Chemical composition

The compounds of aerial parts essential oil of *L. resedifolia *from Algeria are listed in order of their elution on the HP-5MS non-polar column (Table 1). A total of 19 compounds were identified, representing 86.68% of the total oil. The esters made up the largest component of the oil including Dioctyl phthalate (39.84%), Decanoic acid, decyl ester (12.09%) and (E)-2-Heptenoic acid, ethyl ester (5.21%). Aldehydes represent the second largest group (11.45) involving 11-Octadecenal (11.24%) and Heptanal (0.21%).

**Table 1:** Chemical content of essential oils of *L. resedifolia L*

	Compound	RT (min)	Percentage
1	Pentanedioic acid, dimetyl ester	14.56	0.13
2	linalool	26.32	1.45
3	Eucalyptol	29.88	7.31
4	Hexadecanol	31.17	2.82
5	Octanol	32.05	0.87
6	α-Limonene diepoxide	32.13	0.19
7	(Z)-6-Octen-2-one	33.23	0.64
8	Heptanal	36.15	0.21
9	3,4-Dimethylcyclohexanol	36.61	0.13
10	bornyl acetate	36.83	0.19
11	caryophyllene oxide	37.68	1.04
12	1,2-Benzenedicarboxylic acid, butyl octyl ester	38.37	0.22
13	Dibutylphthalate	45.51	2.93
14	(Z)-3-Dodecene,	50.38	0.17
15	Hexanedioic acid, dioctyl ester	56.52	Tr.
16	(*E*)-2-Heptenoic acid, ethyl ester,	60.98	5.21
17	Dioctyl phthalate	61.93	39.84
18	11-Octadecenal	66.81	11.24
19	Decanoic acid, decyl ester	72.39	12.09
	**Total**		**86.68**
	Esters		60.61
	Aldehydes		11.45
	Oxygene monoterpenes		8.95
	Alcohols		03.82
	Oxygene sesquiterpenes		1.04
	Ketones		0.64
	Alkenes		0.17

The monoterpenes represent a relatively low content (8.95%) with eucalyptol as the major constituent (7.31%). A better agreement was found between the oil content of *L. resedifolia *and that of *L. arboresens *as was reported by Cheriti et al. [6]. The slight difference may be due to the geographical location and the harvesting period. It is noteworthy that the results of this study may be considered as the first report on the composition of the essential oils of this endemic species.

### 2.6 Antimicrobial activity

The quantification of antibacterial activity for *L. resedifolia *essential oils was measured by the agar disk diffusion method. The effectiveness of the essential oil is demonstrated by the size of the microorganism growth inhibition zone around the filter paper disk, which is typically expressed as the diameter of the inhibition zone in millimeter. Results obtained in the antibacterial study are shown in Table 2. The results indicated that *S. aureus *was the most sensitive strain to the oil of *L. resedifolia *with the strongest inhibition zone (37 mm) and a MIC value of 0.09 mg/mL. The strains *S. intermedius, K. pneumoniae, S. pyogenes *and *P. mirabilis *were found to be fairly sensitive with inhibition zones of 29, 27, 23, and 20 mm, respectively. Modest activities were observed against *E. coli *and *Pseudomonas aerugenosa *with inhibition zones of 15 and 12 mm. Against *S. intermedius, K. pneumoniae, S. pyogenes, P. mirabilis, E. coli *and, *P. aerugenosa*, the oils showed MIC values of 0.13, 0.21, 0.35, 0.47, 0.54, and 0.69 mg/mL, respectively.

**Table 2:** Inhibition zone diameter (mm)

Microorganisms	Disc diffusion assay (inhibition zone mm)	MIC (mg/mL)
*S. aureus*	37	0.09
*S. intermedius*	29	0.13
*K. pneumoniae*	27	0.21
*S. pyogenes*	23	0.35
*P. mirabilis*	20	0.47
*E. coli*	15	0.54
*P. aerugenosa*	12	0.69

## 3. Conclusions

The chemical analyses by GC/MS allowed the identification of 86.68% of the total volatile products for *L. resedifolia *and 19 volatile compounds. A major constituent in visible parts was Dioctyl phthalate (39.84%) and the yield of essential oils was 0.9%. These extracts reveal *in vitro *antibacterial activity on the studied bacterial, confirmed by the inhibition zone diameter ranging from 11 to 37 mm and a MIC value between 0.09 and 0.69 depending on the microorganism being tested. Antibacterial activities of these essential oils were due to abundance of overall chemical constituents. The antibacterial activity besides several biological activities can be used in place of costly antibiotics for effective control of the food pathogens.

## Competing interests

The authors declare that they have no competing interests.
